# Rs7911488 modified the efficacy of capecitabine-based therapy in colon cancer through altering miR-1307-3p and TYMS expression

**DOI:** 10.18632/oncotarget.19670

**Published:** 2017-07-28

**Authors:** Qi Chen, Yong Mao, Fanyi Meng, Lei Wang, Hongjian Zhang, Weipeng Wang, Dong Hua

**Affiliations:** ^1^ Department of Medical Oncology, Institute of Cancer, Affiliated Hospital of Jiangnan University, The Fourth People’s Hospital of Wuxi, Wuxi 214062, China; ^2^ Center for Drug Metabolism and Pharmacokinetics, College of Pharmaceutical Sciences, Soochow University, Suzhou 215123, China; ^3^ Department of Pharmacy, Jiangsu Cancer Hospital, Nanjing 210000, China

**Keywords:** colon cancer, capecitabine, polymorphism, TYMS, miR-1307-3p

## Abstract

Capecitabine is an orally administered prodrug of 5-fluouracil (5-FU) and is used in first-line treatment of metastatic colorectal cancer. Studies have demonstrated that polymorphisms in 5-FU related ADME genes are associated with the efficacy of capecitabine. However, the relationship between the polymorphisms within the microRNA precursors and the efficacy of capecitabine is still largely unknown. We detected six polymorphisms in 274 colon cancer patients and statistically analyzed the association of the genotypes with the efficacy of capecitabine-based chemotherapy. The mechanisms underlying the effect of genotypes on the efficacy of capecitabine were also studied. We identified a polymorphism rs7911488 T>C in pre-miR-1307 to be significantly associated with the efficacy of capecitabine chemotherapy in colon cancer patients. The response rates of capecitabine chemotherapy for the patients with TT, TC, and CC genotypes were 44.35% (55/124), 51.33% (58/113), and 24.32% (9/37), respectively. In the C-allelic patients, miR-1307-3p is down-regulated and TYMS, a direct target of miR-1307-3p, is over-expressed, which leads to insensitivity of cancer cells to capecitabine chemotherapy. The cancer cells with rs7911488 C allele were further observed to be resistant to 5-FU treatment *in vitro* and *in vivo*. Our findings show that rs7911488 C-allelic pre-miR-1307 leads to attenuated miR-1307-3p and elevated TYMS, thus insensitive to capecitabine chemotherapy in colon cancer.

## INTRODUCTION

Capecitabine (Xeloda, Roche), an oral 5-fluorouracil (5-FU) pro-drug, is commonly given to patients with colorectal cancer (CRC). Capecitabine is activated to 5-FU, which is converted to metabolites and incorporated into DNA and RNA to inhibit their synthesis [1]. 5-FU has been widely used since the 1960s as first-line therapy for CRC both in the adjuvant and palliative setting. It remains the backbone of many combination chemotherapy regimens [2]. However, the overall efficacy rate of 5-FU/capecitabine based therapy is only 24.8% [3]. That means a number of patients will not benefit from the treatment of capecitabine. Moreover, the effective treatments might be missed and the treatment costs will be wasted.

The biochemical pathway of capecitabine activation and subsequent 5-FU action and degradation has been well established. There are 25 candidate genes in which variation might affect the efficacy and toxicity of capecitabine chemotherapy [1, 4–7]. Upon absorption in the gut, capecitabine is partially converted to 5-FU in the liver, and then is preferentially converted to 5-FU at the tumor site. 5-FU is further activated in the tumor to cytotoxic compound FdUMP that inhibits DNA synthesis by competing with nucleotide precursor for binding with thymidylate synthase (TYMS).

Numerous studies have provided evidences to prove that the interpatient differences in efficacy are related to various factors, such as age, gender, local clinical practice, diet, and genetic background. Over a decade of publications exists regarding inherited genetic biomarkers of capecitabine efficacy, but only a few of polymorphisms and rare genetic variants have been identified with clinic significance to be associated with capecitabine [6–8]. These include two common polymorphisms in TYMS (5’-UTR 2R/3R and 3’-UTR 6 bp ins-del) and rare functionally deleterious variants rs3918290 (DPYD*2A), rs55886062 (DPYD*13), and rs67376798 (2846T>A) [7, 9, 10]. The TYMS genotypes predict TYMS mRNA expression, response and toxicity to 5-FU treatment in metastasized colon cancer. Patients with the 3R/3R genotype have significantly less response and toxicity to 5-FU-based chemotherapy when compared with the 2R/2R genotype [11]. These variants are potentially useful, but suboptimal, for the prediction of efficacy in clinical practice. Furthermore, there is only limited evidence that genetic variants are generalizable as predictors of efficacy across 5-FU regimens [7].

MicroRNA (miRNA), a small RNA with about 23 nts, has been well documented as a key regulator in gene expression. Numerous studies have showed that polymorphisms in the miRNA precursors (pre-miRNA) alter the expression of miRNAs and/or the binding between miRNAs and their target genes, and consequently the expression of targets [12]. Recently, more and more polymorphisms in the pre-miRNAs have been discovered to be evidently related to the efficacy and/or toxicity of chemotherapy in cancers [13]. For instance, a miR-200b/200c/429-binding site polymorphism in the AP-2α gene is associated with cisplatin resistance [14]. Two polymorphisms (rs4919510 in miR-608 and rs213210 in miR-219-1) are correlated to the efficacy of 5-FU [15]. A variant in the KRAS gene has been identified as a biomarker of poor outcome and platinum chemotherapy resistance in ovarian cancer [16]. However, the relationships between the polymorphisms in the pre-miRNAs and the efficacy and toxicity of capecitabine-based therapy in colon cancer are still largely unclear.

In this study, to investigate the associations of the polymorphisms in pre-miRNAs with the efficacy and toxicity of capecitabine-based chemotherapy, the genotypes of six polymorphisms were determined in 274 colon cancer patients. The mechanisms underlying the effect of genotypes on the efficacy of capecitabine were also studied.

## RESULTS

### The colon cancer patients with rs7911488 C allele are insensitive to capecitabine

To investigate the correlation between polymorphisms in pre-miRNAs and the efficacy of capecitabine chemotherapy, ninety one polymorphisms and their allele frequencies were obtained from the online databases miRNASNP and NCBI dbSNP (Supplementary Table 1). Among these polymorphisms, the minor allele frequencies of 10 polymorphisms were more than 0.1 (Supplementary Table 1). We detected these polymorphisms in 274 colon cancer patients by using MassARRAY SNP Genotyping method. Six of the 10 polymorphisms were successfully detected, but 4 polymorphisms were not detected due to potential interaction among primers and/or unspecific amplification. The typical genotyping results of the 6 polymorphisms are shown in Supplementary Figure 1. As shown in Table 1, the polymorphism rs7911488 in pre-miR-1307 was found to be significantly correlated to the efficacy of capecitabine chemotherapy. Compared with the TT homozygotes, the CC homozygotes were significantly less sensitive to capecitabine treatment (24.32% vs 44.35%; Table 1). We also analyzed the association of genotypes with the toxicity of capecitabine chemotherapy. We found that the occurrence rate of toxicity in the patients with CC genotype was much higher than that in the TT homozygous patients, even though the difference is not statistically significant (81.08% vs 64.52%; *P*=0.070; Supplementary Table 2). There was no correlation of the genotypes of rs2910164, rs3746444, rs6505162, rs12402181, and rs12416605 with the efficacy and toxicity of capecitabine chemotherapy (Supplementary Table 2 and 3).

**Table 1:** The association of rs7911488 with the efficacy of capecitabine therapy in colon cancer patients

Genotype	Response^a^		Response rate (%)	OR (95% CI) ^b^	*P*-value
	PD+SD	PR+CR			
T/T	69	55	44.35	1.00	
T/C	55	58	51.33	0.76 (0.45-1.26)	0.300
C/C	28	9	24.32	2.48 (1.08-5.69)	**0.035**

^a^PD, progressive disease; SD, stable disease; PR, partial response; CR, complete response.

^b^OR, odds ratio; CI, confidence interval.

### The rs7911488 C allele is related to low expression of miR-1307-3p

We and others have found that the rs7911488 genotypes are significantly associated with the expression of miR-1307-3p [17–19]. To further confirm the relationship between rs7911488 genotypes and the expression of miR-1307-3p, we generated stable gene transduction cell lines for expression of rs7911488 T-allelic or C-allelic pre-miR-1307 (SW480-T or SW480-C) and then determined the expression level of mature miR-1307-3p in them. We found that the level of miR-1307-3p in the SW480-C cells was predominantly lower than that in the SW480-T cells (Supplementary Figure 2A), indicating that the C-allelic pre-miR-1307 yields less miR-1307-3p than the T-allelic ones. This was also supported by the results from 1000 Genome projects (Supplementary Figure 2B).

### Low-expression of miR-1307-3p results in up-regulation of TYMS

**Figure 1:**
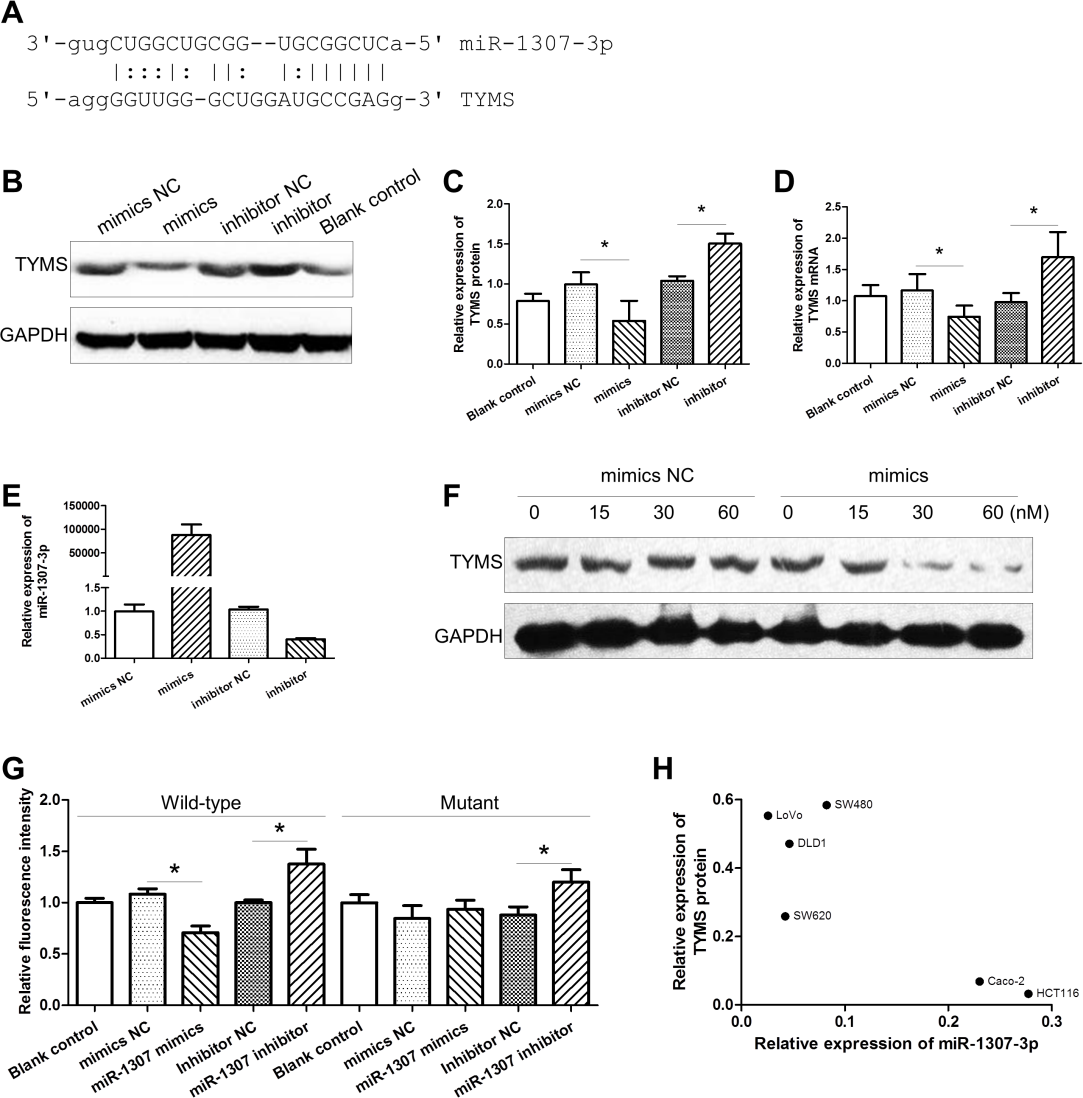
The effect of miR-1307-3p on the expression of TYMS **(A)** The sequence of *TYMS* 3’-UTR harbors putative binding-site for miR-1307-3p. **(B and C)** The effect of miR-1307-3p on the expression of TYMS protein in CRC cells. **(D)** The effect of miR-1307-3p on the expression of TYMS mRNA in CRC cells. **(E)** The expression of miR-1307-3p in CRC cells transfected with miR-1307-3p mimics or inhibitors. **(F)** The effect of various doses of miR-1307-3p on the expression of TYMS protein in CRC cells. **(G)** The effect of miR-1307-3p on the expression of wildtype and mutant TYMS/3’-UTR/pGL3 constructs. **(H)** The negative correlation between the expression of miR-1307-3p and the level of TYMS protein in CRC cells. NC, negative control; *, *P*<0.05.

To investigate the regulative role of miR-1307-3p on the expression of genes that might impact capecitabine efficacy, we used online software miRanda and TargetScan to predict the potential targets of miR-1307-3p. We found that potential binding-sites for miR-1307-3p on the 3’-UTR of TYMS, an action target of capecitabine (Figure 1A). Upon transfection into CRC cells, miR-1307-3p mimics led to overexpression of mature miR-1307-3p and low-expression of TYMS protein and mRNA, while miR-1307-3p inhibitors decreased the expression of mature miR-1307-3p and increased the expression of TYMS protein and mRNA (Figure 1B-1E). Moreover, we found that the effect of miR-1307-3p on the expression of TYMS protein was in a dose-dependent manner (Figure 1F). To evaluate the binding effect of miR-1307-3p with TYMS, we constructed wildtype and mutant TYMS/3’-UTR/pGL3 plasmids and co-transfected them with miR-1307-3p mimics or inhibitors into HCT-116 cells. We found that the expression of wildtype TYMS/3’-UTR/pGL3 plasmids was significantly inhibited by miR-1307-3p mimics and was enhanced by miR-1307-3p inhibitors (Figure 1G). However, miR-1307-3p mimics, except for miR-1307-3p inhibitors, showed no effect on the expression of mutant TYMS/3’-UTR/pGL3 plasmids, indicating that miR-1307-3p can inhibit the expression of TYMS by binding to the 3’-UTR of TYMS gene. We also detected the expression of miR-1307-3p and TYMS protein in six CRC cell lines, and found that the expression of TYMS protein was inversely correlated to miR-1307-3p (Figure 1H).

### The rs7911488 C-allelic cells are insensitive to 5-FU

**Figure 2:**
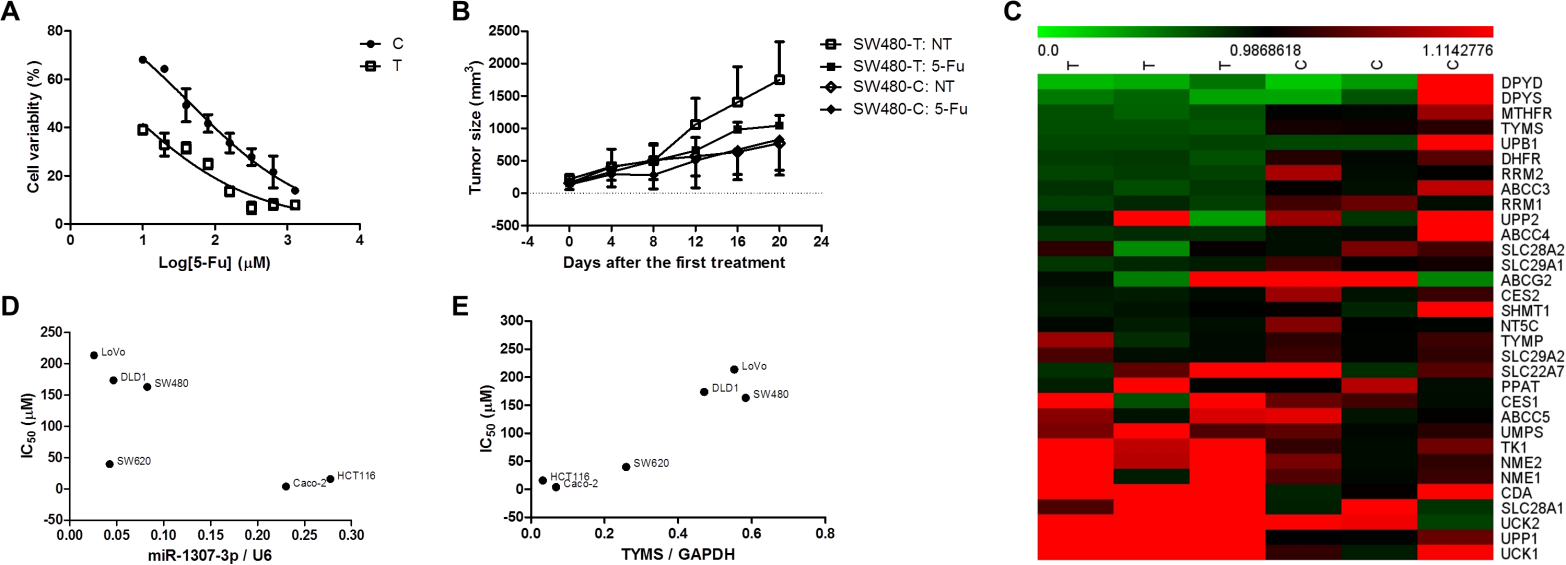
The effect of rs7911488 genotypes on the efficacy of 5-FU chemotherapy **(A)** The inhibitory role of 5-FU on the proliferation of SW480-C and SW480-T cells *in vitro*. **(B)** The inhibitory role of 5-FU on the growth of SW480-C and SW480-T xenografts in nude mice (n=5). **(C)** The expression of 5-FU ADME genes in the SW480-T and SW480-C cells. **(D)** The negative relationship between the expression of miR-1307-3p and *IC*_50_ values of 5-FU in CRC cells. **(E)** The positive relationship between the expression of TYMS protein and *IC*_50_ values of 5-FU in CRC cells.

We evaluated the effect of 5-FU on the proliferation of SW480-C and SW480-T cells. We found that SW480-C cells were evidently less sensitive to 5-FU than SW480-T cells (with *IC*_50_ values of 45.48±1.08 μM *vs* 4.885±1.220 μM; Figure 2A). We subcutaneously grafted SW480-C and SW480-T cells into the lower back of nude mice. On day 21 after xenograft implantation, a group of tumor-bearing mice received intraperitoneally (i.p.) injections of 20 mg/kg of 5-FU once every 2 days. As shown in Figure 2B, upon the treatment of 5-FU, the SW480-C xenografts were out of control, but the growth of SW480-T xenografts were prevented. These findings suggest that the SW480-C tumors are less sensitive to 5-FU than the SW480-T tumors. To explore the mechanism under the difference in the sensitivity of SW480-C and SW480-T cells to 5-FU, we determined the expression of genes in these two types of cells by using microarray gene expression analysis. We found that the expression levels of DPYD, DPYS, TYMS, and UPB1 were significantly lower in the SW480-T cells, but the expression levels of CDA, SLC28A1, UCK1, UCK2, and UPP1 were apparently higher in the SW480-T cells, as compared with those in the SW480-C cells (Figure 2C). In addition, we investigated the effect of 5-FU on the proliferation of the other CRC cells including Caco-2, DLD1, HCT-116, LoVo, SW480, and SW620 (Supplementary Figure 3). Interestingly, we found that the *IC*_50_ values were positively correlated to the expression of miR-1307-3p (Figure 2D and Supplementary Figure 4) and were reversely correlated to the expression of TYMS protein in these cell lines (Figure 2E and Supplementary Figure 5).

## DISCUSSION

In this study, we have investigated the relationship between polymorphisms within pre-miRNAs and the efficacy of capecitabine. Our results reveal a significant correlation of rs7911488 T>C in pre-miR-1307 with the efficacy of capecitabine in colon cancer patients. The response rate of the capecitabine-based treatment is the highest in the patients with rs7911488 TC genotype and is the lowest in those with CC genotype. Mechanistic studies demonstrate that the rs7911488 C allele leads to low-expression of miR-1307-3p, high-expression of TYMS, and consequently insensitivity of cancer cells to capecitabine-based treatment. These findings discover a novel biomarker for the efficacy of capecitabine.

Twenty years ago, intratumoral TYMS mRNA expression was established as a predictor of efficacy and survival of CRC patients with 5-FU chemotherapy. Leichman et al. showed that low-expression of TYMS mRNA is associated with a long survival and high response rate to 5-FU based treatment. The patients with a TYMS mRNA expression level more than 4.1×10^-3^ will not respond to 5-FU chemotherapy [20]. Increased intratumoral TYMS mRNA expression has been found to be positively related to an increasing number of a 28-bp tandem repeats in the 5'-UTR of TYMS. Recent studies showed that TYMS protein expression in gastrointestinal tumors was also associated with this polymorphism [11, 21]. In this study, we found that the sensitivity of CRC cells to 5-FU was negatively related to the expression of TYMS protein. Since the expression of TYMS mRNA and protein are inhibited by miR-1307-3p, the sensitivity of CRC cells to 5-FU is positively correlated to the expression of miR-1307-3p, indicating that the expression of miR-1307-3p might be regarded as a predictor of efficacy of 5-FU chemotherapy in colon cancer patients.

Statistical analysis results demonstrate that the response rate of capecitabine chemotherapy for the patients with CC genotype is the lowest, then is the TT homozygous individuals, and those with TC genotype is the highest. However, the response rate of CC homozygotes were much lower than the total response rate (24.32% *vs* 44.53%), indicating the CC homozygous patients are less benefited from the capecitabine-based treatment. In addition, the TC heterozygotes have a higher response rate than the TT homozygotes (51.33% *vs* 44.35%), indicating that the TC heterozygotes are more sensitive to capecitabine chemotherapy than the TT homozygotes. This was also confirmed by our *in vitro* experiments. We found that TC heterozygous Caco-2 and HCT-116 cells were more sensitive to 5-FU treatment than the TT homozygous DLD1, LoVo, SW480, and SW620 cells (Supplementary Figure 3).

TYMS, the action target of 5-FU, has been studied intensively for the past decade because of its important role in anti-cancer therapies. Over-expression of TYMS protein has been detected in various human carcinomas including CRC. Up to now, miR-192 [17], miR-215 [18], and miR-433 [22] have been revealed to be involved in the regulation of human TYMS protein. By using software miRanda and TargetScan, we found a conserved binding site for miR-1307-3p in the 3’-UTR of TYMS gene. We then provided evidences to prove that TYMS is a direct target of miR-1307-3p: (i) the expression of both TYMS mRNA and protein in cancer cells were suppressed by miR-1307-3p mimics and elevated by miR-1307-3p inhibitors; (ii) the luciferase report assays demonstrated that miR-1307-3p mimics inhibited the expression of TYMS by binding to the 3’-UTR of TYMS; (iii) the expression levels of TYMS protein in cancer cell lines were negatively correlated to the expression levels of miR-1307-3p; and (iv) microarray data indicated that the expression levels of TYMS mRNA in the SW480-T cells (with high-expression of miR-1307-3p) were obviously lower than those in the SW480-C cells (with low-expression of miR-1307-3p). In addition, ABCC3 and UCK1 were predicted to be potential targets of miR-1307-3p (Supplementary Figure 6A). However, the luciferase report assays showed that these genes were not regulated by miR-1307-3p (Supplementary Figure 6B).

The biochemical pathway of capecitabine *in vivo* is well established [1]. Capecitabine is first uptake by the transporters SLC28A1 and SLC28A2, and then is transferred to 5-FU by enzymes including cytidine deaminase (CDA) and uridine phosphorylase (UPP1). 5-FU is partially converted to FdUMP to inhibit DNA synthesis by binding with TYMS. Besides, a section of 5-FU is metabolized by enzymes including UPP1 and uridine cytidine kinases (UCK1 and UCK2) into FUTP, which inhibits the synthesis of RNA by incorporating into RNA. However, about 85% of 5-FU is inactivated by enzymes dihydropyrimidine dehydrogenase (DPYD), dihydropyrimidine (DPYS), and β-ureidopropionase (UPB1). Interestingly, in this study we found that the expression levels of DPYD, DPYS, TYMS, and UPB1 were significantly lower, but the expression levels of CDA, SLC28A1, UCK1, UCK2, and UPP1 were apparently higher in the fluorouracil-sensitive SW480-T cells, as compared with the fluorouracil-insensitive SW480-C cells. These findings demonstrate that miR-1307-3p might alter the sensitivity of cancer cells to capecitabine through regulating the expression of these enzymes and/or transporters, though the regulatory mechanisms need to be further investigated.

In summary, our study provides clear evidences of genetic linkage between rs7911488 in pre-miR-1307 and the efficacy of capecitabine-based treatment in colon cancer patients. To our knowledge, this study is the first to report on the association of this polymorphism with the response of capecitabine. The C allele of this polymorphism leads to low-expression of miR-1307-3p, elevated expression of TYMS and insensitive to capecitabine chemotherapy. The present findings are of clinical relevance in understanding the role of polymorphisms in pre-miRNAs in chemotherapy.

## MATERIALS AND METHODS

### Patients

A total of 274 eligible patients with colon cancer were assigned to 3 weekly cycles of capecitabine (1,000 mg/m^2^ bid. for 14 days) and oxaliplatin (130 mg/m^2^ on day 1). No previous chemotherapy or adjuvant chemotherapy for metastatic disease was allowed. Adverse events including nausea and vomiting, myelosuppression, liver dysfunction, diarrhea, and neurotoxicity were recorded. The doses of capecitabine were reduced in patients with adverse reactions. Tumor evaluation was done every 9 weeks according to RECIST (Response Evaluation Criteria in Solid Tumors) Version 1.0. The efficacy of chemotherapy including complete response (CR), partial response (PR), stable disease (SD), and progressive disease (PD) were recorded. The characteristics of the patients were listed in Supplementary Table 4. The study was approved by the institutional review boards of the Affiliated Hospital of Jiangnan University. All patients provided written informed consent before study entry. Blood samples and germline DNA were obtained from the eligible patients prior to start of therapy. All clinical results were blinded by genotype.

### Genotyping

The polymorphisms in the pre-miRNAs and their allele frequencies were obtained from the online databases of miRNASNP 2.0 (www.bioguo.org/miRNASNP2) and NCBI dbSNP (https://www.ncbi.nlm.nih.gov/projects/SNP/), respectively (Supplementary Table 1). The polymorphisms with minor allele frequencies (MAF) ≥ 10% were detected by using a MassARRAY SNP Genotyping platform (SEQUENOM, In.) as described previously [17]. In brief, the DNA fragments containing polymorphisms were amplified by PCR. Then single-base extensions were performed on the PCR amplificons. Next, the single-base extension products were desalted and then transferred to the MassARRAY SpectroCHIP chips for mass spectra analysis. The genotypes were read on the mass spectrums. The association of genotypes with the efficacy and toxicity of capecitabine chemotherapy were analyzed by using SPSS11.5.

### Quantitative real-time PCR

The CRC cell lines including Caco-2, DLD-1, HCT116, LoVo, SW480, and SW620 were cultured as described before [17]. Total RNAs from the CRC cells and those transfected with 50 nM miR-1307-3p mimics or inhibitors (GenePharma, Shanghai, China) for 72 h were isolated using TRIzol (Invitrogen). The RNAs were then reversely transcripted using M-MuLV reverse transcriptase (MBI) and stem-loop primers for miR-1307-3p or random primer for TYMS and GAPDH. Quantitative real-time PCRs (qPCRs) were conducted in 20-μL reaction containing each sequence-specific primer (Supplementary Table 5) and quantitative RT-PCR master mix (Takara). The qPCRs were carried out on the CFX96 Touch™ real-time PCR system (Bio-Rad). The expression levels were calculated by using the comparative threshold cycle (Ct) method with the formula 2^-Δ ΔCt^.

### Immunoblotting

The CRC cells and those transfected with 50 nM miR-1307-3p mimics or inhibitors for 72 h were lysed using RIPA buffer (Rockland Immunochemical Inc., USA) with the complete protease inhibitor cocktail (Roche). The protein concentration was determined using the Pierce BCA Protein Assay Kit (Thermo). Then the proteins were separated on SDS-PAGE gel and electro-transferred onto PVDF membranes (Bio-Rad). The membranes were incubated with TYMS or GAPDH mouse monoclonal antibody, and subsequently with a peroxidase goat anti-mouse antibody (Santa Cruz Biotech Inc., USA). The proteins on the membranes were visualized with Clarity Western ECL substrates (Bio-Rad) in ChemiDoc™ MP Imaging System (Bio-Rad).

### Luciferase reporter assay

The potential targets of the miRNAs were predicted by using online softwares miRanda (http://www.microrna.org/microrna/home.do) and TargetScan (http://www.targetscan.org/mamm_31/). As listed in Supplementary Table 1, TYMS, UCK1, and ABCC3 were predicted to be potential targets of miR-1307-3p. Thus luciferase reporter assays were performed to evaluate the regulatory role of miR-1307-3p in the expression of these target genes as previously described [17]. Briefly, the wildtype pGL3 constructs containing each of the 3’-UTRs of TYMS, UCK1, and ABCC3 genes were generated by amplifying the 3’-UTRs with the primers in Supplementary Table 5. The amplification products were cloned into the downstream of the pGL3-Control vectors (Promega) using *Xba*I and *Hpa*l endonucleases (New England Biolabs). The mutant TYMS/3’-UTR/pGL3 constructs were generated by mutating the binding-site of “GCCGAG” for miR-1307-3p to “CGGCTC” using site-directed mutagenesis PCR method with the primers in Supplementary Table 5. Positive clones were selected by sequence-specific PCR, restriction enzymes digestion, and DNA sequencing method. After that, 50 nM miR-1307-3p mimics or inhibitors were co-transfected with 200 ng of the wildtype or mutant 3’-UTR/pGL3 constructs and 200 ng of pRL-TK plasmids (Promega) into HCT-116 cells by using lipofectamine 2000 (Invitrogen). After incubating for 24 h, the activities of luciferase in cells were detected by using the dual-luciferase reporter assay system (Promega). Each transfection was performed in triplicate.

### Stable gene transduction cell lines

The expression vectors for rs7911488 T-allelic or C-allelic pre-miR-1307 have been described previously [17]. The resulting expression vectors were transfected into SW480 cells and stable clones (SW480-T and SW480-C) were established by neomycin selection. The stable clones were evaluated by flow cytometry assays.

### Cell viability assay

The effects of 5-FU on the proliferation of Caco-2, DLD1, HCT-116, LoVo, SW480, SW620, SW480-T and SW480-C cells were determined by using MTT assays. The cells were seeded in 96-well plates and were incubated with 6.25, 12.5, 25.0, 50.0, or 100 μM of 5-FU for 72 h. Then the cell growth was measured using MTT analysis with the M3 SpectraMax microplate reader. The *IC*_50_ values of 5-FU were estimated according to the absorbance.

### Xenografts

The animal experiments were performed in accordance with currently prescribed guidelines and under a protocol approved by the Institutional Animal Care and Use Committee at the Affiliated Hospital of Jiangnan University. SW480-T and SW480-C cells were collected, counted, and mixed with Matrigel (BD Biosciences) in a 1:1 ratio by volume. Cells (5×10^6^) in 100 μL of medium/Matrigel solution were subcutaneously (s.c.) injected in the lower back region of male nude mice (SLAC int., Shanghai, China). The mice were randomly divided into four groups with five mice in each group when the tumors have reached a volume of 150-200 mm^3^. Twenty mg/kg of 5-FU were intraperitoneally (i.p.) administered once every 2 days. Tumor volumes were measured every 4 days and were calculated by the formula (width^2^×length/2).

### Microarray analysis

The expression of 32 genes, which participated in the transportation and metabolization of capecitabine (https://www.pharmgkb.org/pathway/PA150653776), in the SW480-T and SW480-C cells were determined by using microarray-based gene expression analysis. The total RNA samples were extracted from SW480-T or SW480-C cells in triplicate, and were quantified by the NanoDrop ND-2000 (Thermo Scientific). The RNA integrity was assessed using Agilent Bioanalyzer 2100 (Agilent). The sample labeling, microarray hybridization and washing were performed according to the manufacturer’s standard protocols. Briefly, total RNA were transcribed to double strand cDNA, and then were synthesized into cRNA with cyanine-3-CTP. The labeled cRNAs were hybridized onto the microarray (Agilent SurePrint G3 Human Gene Expression v2). After washing, the arrays were read by a scanner (G2505C; Agilent). Feature Extraction software (version10.7.1.1; Agilent) was used to analyze array images and Genespring (version13.1; Agilent) were employed to finish the basic analysis with the raw data.

## SUPPLEMENTARY MATERIALS FIGURES AND TABLES




