# Supplementary material for: Rs7911488 modified the efficacy of capecitabine-based therapy in colon cancer through altering miR-1307-3p and TYMS expression
Source: Oncotarget. 2017 Jul 28;8(43):74312–9. doi: 10.18632/oncotarget.19670 (PMC5650342; doi:10.18632/oncotarget.19670)
Supplement: Supplementary file 1 [file oncotarget-08-74312-s001.pdf]

Rs7911488 modified the efficacy of capecitabine-based therapy in colon cancer through altering miR-1307-3p and TYMS expression

SUPPLEMENTARY MATERIALS

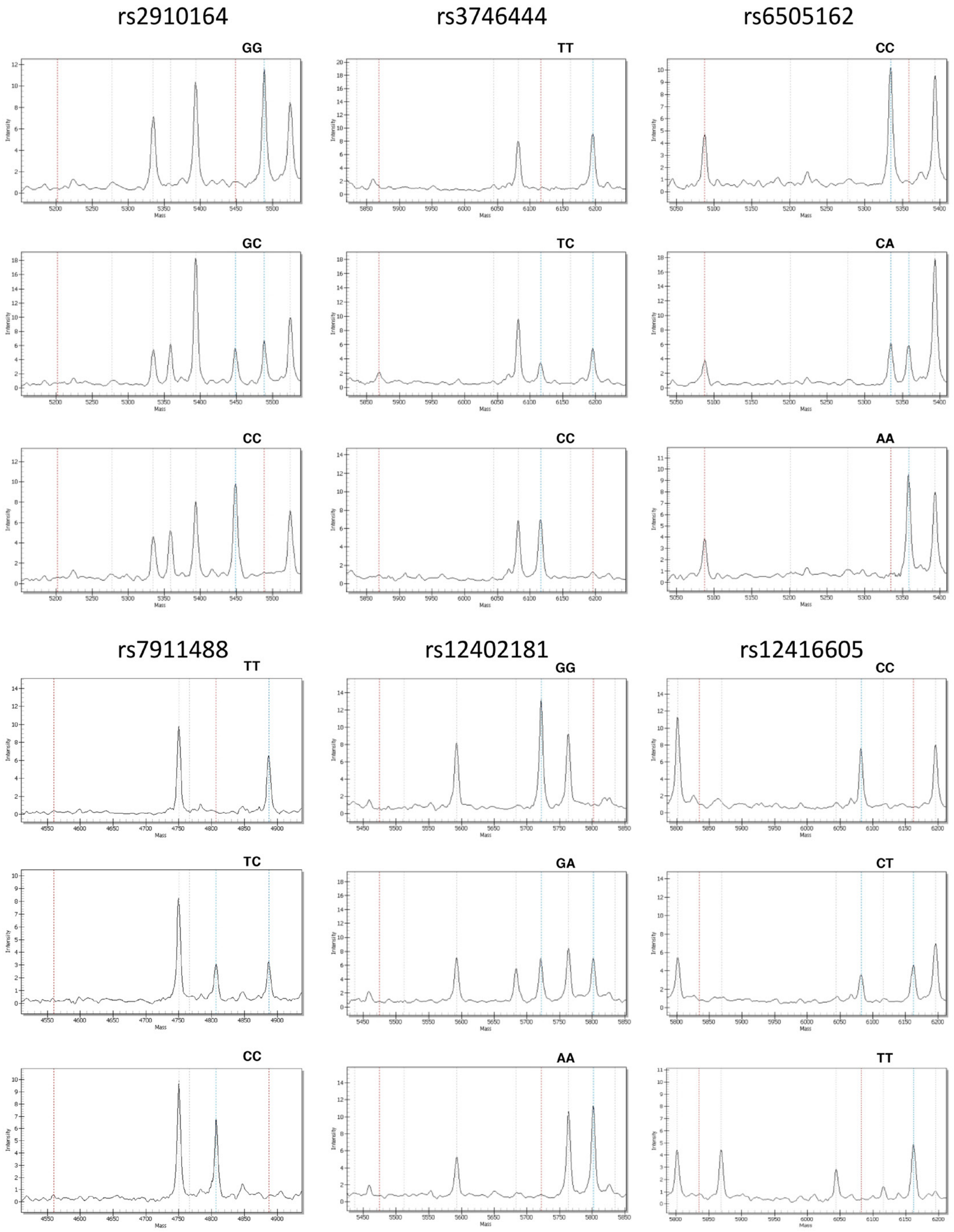

Supplementary Figure 1: The typical genotyping results of rs2910164, rs3746444, rs6505162, rs7911488, rs12402181, and rs12416605.

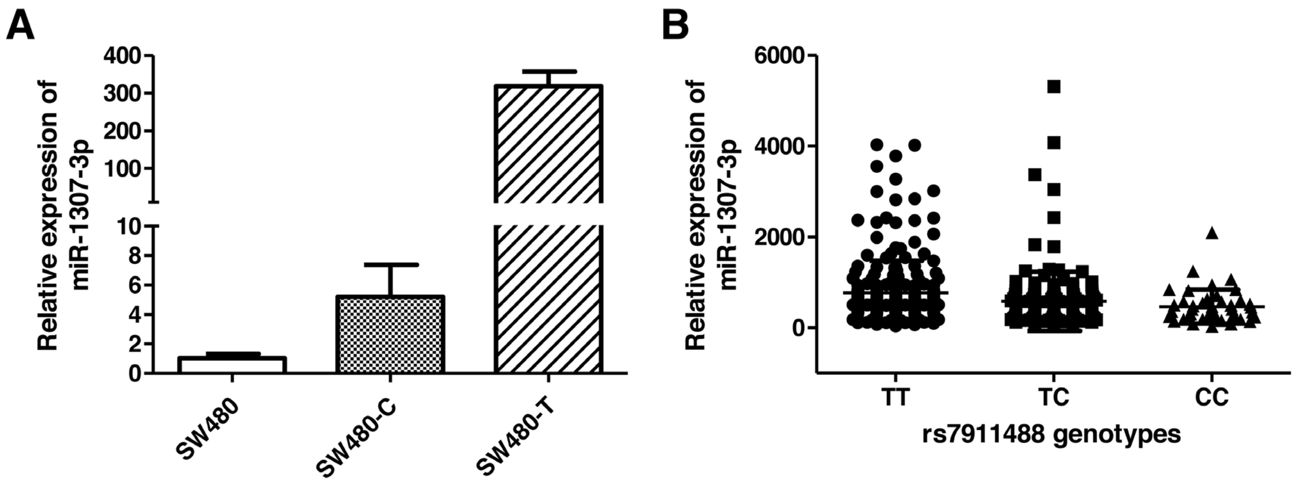

Supplementary Figure 2: The expression of miR-1307-3p in cancer cells. **(A)** The expression of miR-1307-3p in SW480, SW480-C, and SW480-T cells. **(B)** The relationship between the expression of miR-1307-3p and rs7911488 genotypes in 436 samples from 1000 Genome projects (http://www.internationalgenome.org/).

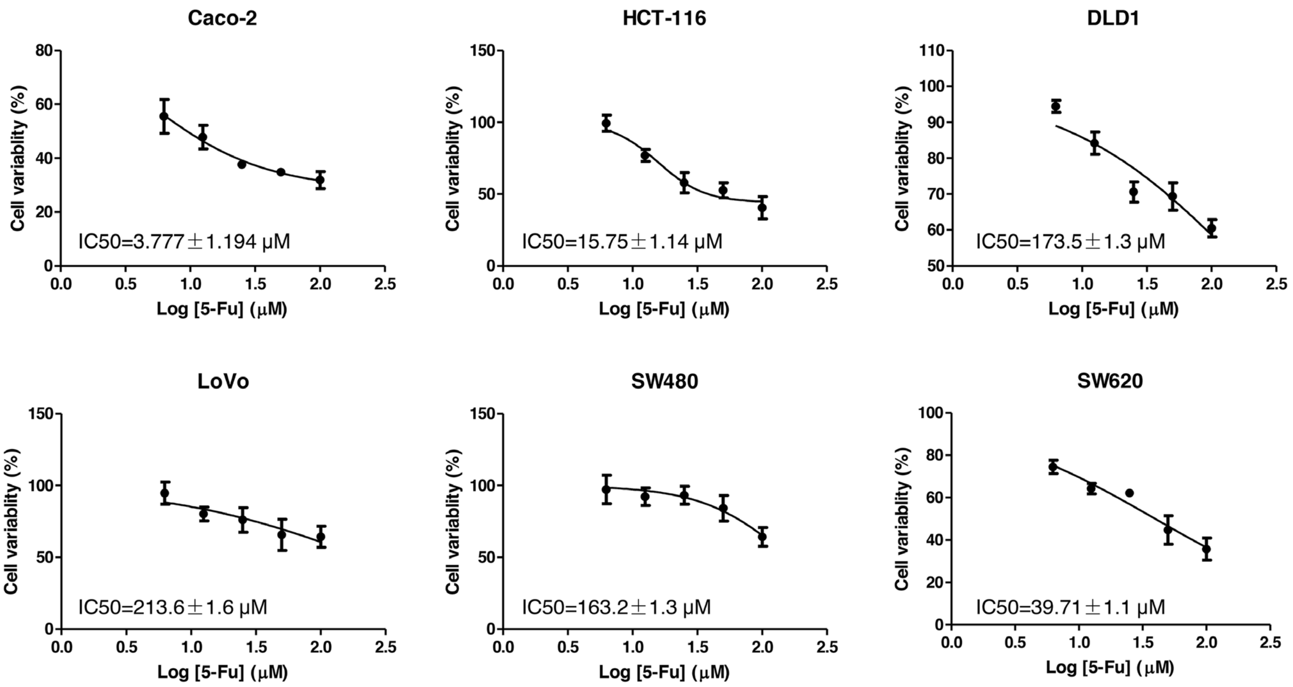

Supplementary Figure 3: The effect of 5-FU on the proliferation of CRC cell lines.

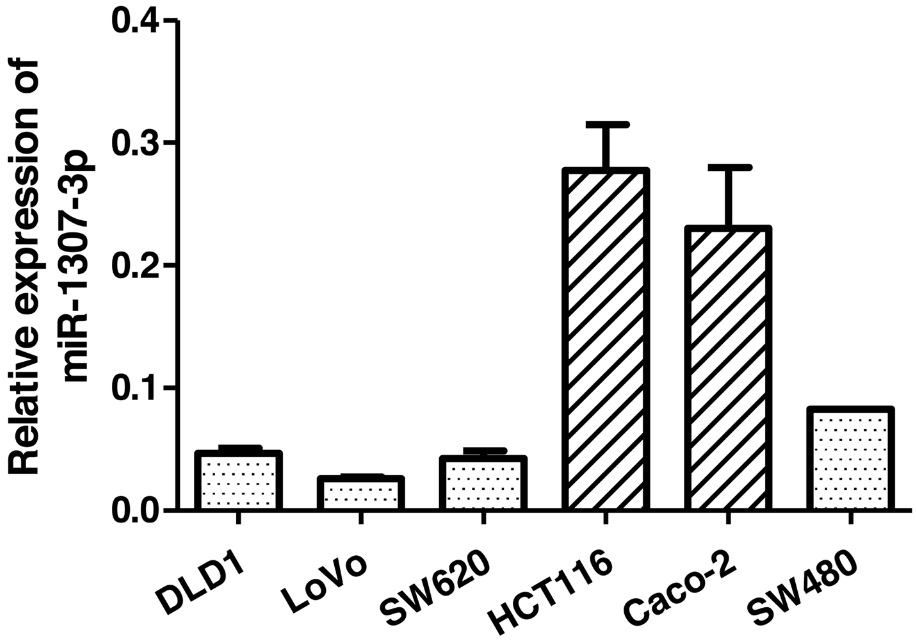

Supplementary Figure 4: The expression of miR-1307-3p in CRC cell lines.

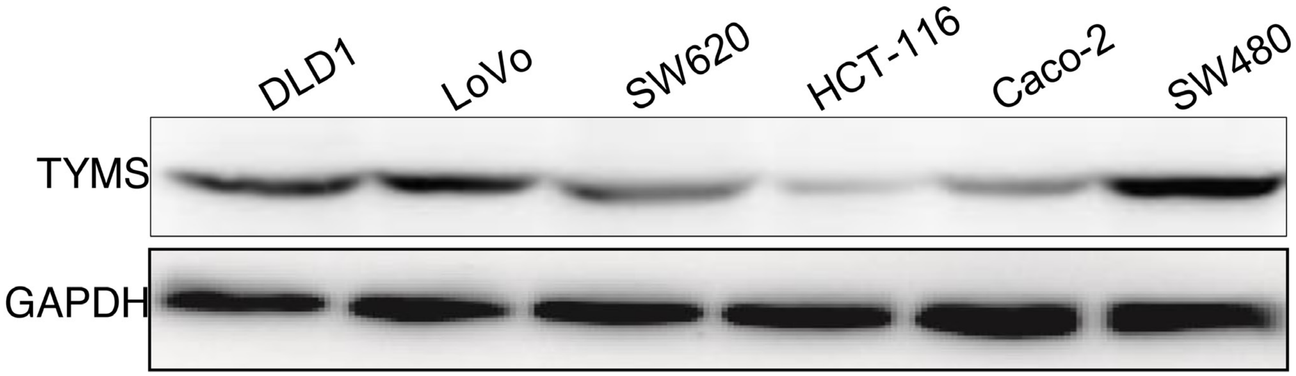

Supplementary Figure 5: The expression of TYMS protein in CRC cell lines.

A

```
938:5'-ugugagcacaaagaaGCCGAGa-3' ABCC3
                      ||||||
    3'-gugcuggcugcgguGCGGCUCa-5' miR-1307-3p
                     :||||||
  1:5' ---------ggggcUGCCGAGc-3' UCK1
```

B

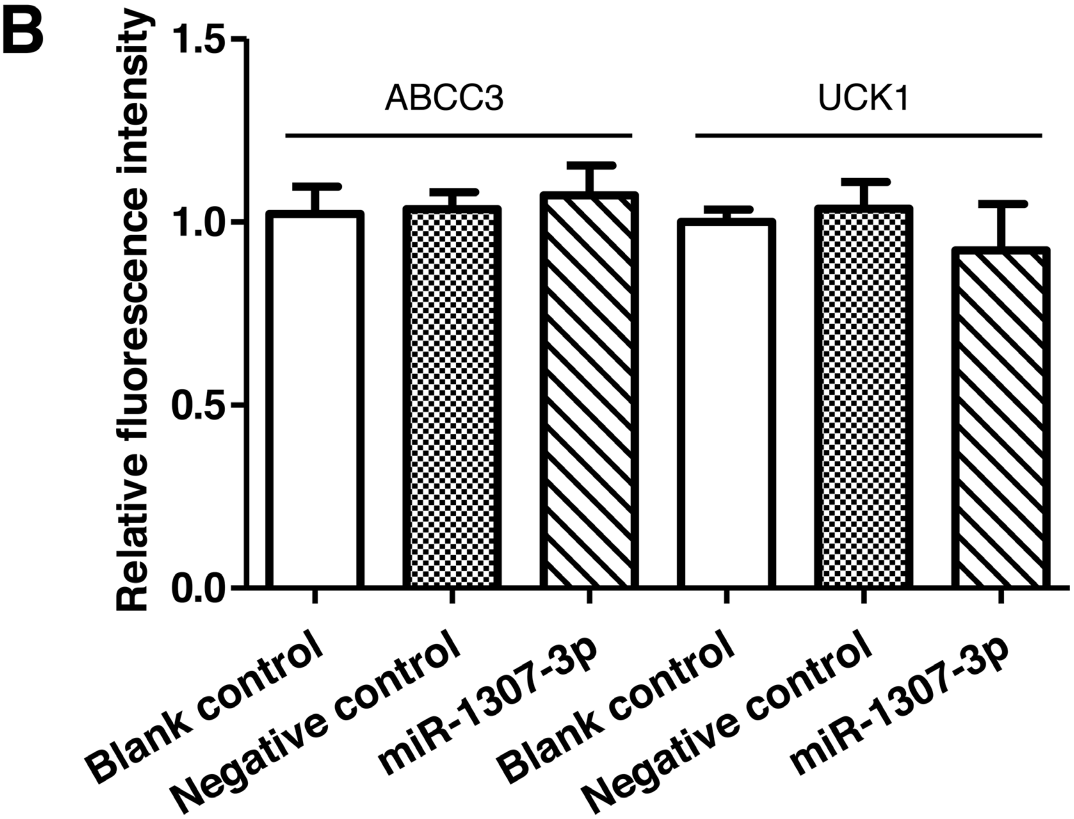

Supplementary Figure 6: Luciferase reporter assays for testing the effect of miR-1307-3p on the expression of ABCC3/3'-UTR/pGL3 and UCK1/3'-UTR/pGL3 constructs.

Supplementary Table 1: The polymorphisms in the pre-miRNAs and the predicted target genes

See Supplementary File 1

Supplementary Table 2: The association of miRSNP with the toxicity of capecitabine therapy in advanced colon cancer

SNP	Genotype	Toxicity		Occurrence rate (%)	OR (95% CI) [a]	*P*-value
		No	Yes			
rs2910164	G/G	31	72	69.90		
	G/C	37	86	69.92	1.00 (0.57-1.77)	1.000
	C/C	18	30	62.50	1.39 (0.68-2.86)	0.456
rs3746444	T/T	56	100	64.10		
	C/T	26	81	75.70	0.57 (0.33-0.99)	0.058
	C/C	4	7	63.64	1.02 (0.29-3.64)	1.000
rs6505162	C/C	57	129	69.35		
	C/A	25	48	65.75	1.18 (0.66-2.10)	0.656
	A/A	4	11	73.33	0.82 (0.25-2.69)	1.000
rs7911488	T/T	44	80	64.52		
	T/C	35	78	69.03	0.82 (0.47-1.40)	0.492
	C/C	7	30	81.08	0.42 (0.17-1.04)	0.070
rs12402181	G/G	29	70	70.71		
	G/A	46	93	66.91	1.19 (0.68-2.09)	0.573
	A/A	11	25	69.44	1.06 (0.46-2.44)	1.000
rs12416605	C/C	69	154	69.06		
	C/T	17	32	65.31	1.19 (0.62-2.28)	0.614
	T/T	0	2	100.0	1.45 (1.33-1.58)	1.000

[a] OR, odds ratio; CI, confidence interval.

Supplementary Table 3: The association of SNPs with the efficacy of capecitabine therapy in advanced colon cancer

SNP	Genotype	Response [a]		Response rate (%)	OR (95% CI) [b]	*P*-value
		PD+SD	PR+CR			
rs2910164	G/G	63	40	38.83		
	G/C	68	55	44.72	0.79 (0.46-1.34)	0.418
	C/C	21	27	56.25	0.49 (0.25-0.99)	0.054
rs3746444	T/T	86	70	44.87		
	C/T	60	47	43.93	1.04 (0.63-1.71)	0.900
	C/C	6	5	45.45	0.98 (0.29-3.34)	1.000
rs6505162	C/C	109	77	41.40		
	C/A	33	40	54.79	0.58 (0.34-1.01)	0.054
	A/A	10	5	33.33	1.41 (0.46-4.30)	0.597
rs12402181	G/G	50	49	49.49		
	G/A	82	57	41.01	1.41 (0.84-2.37)	0.234
	A/A	20	16	44.44	1.23 (0.57-2.64)	0.698
rs12416605	C/C	126	97	43.50		
	C/T	25	24	48.98	0.80 (0.43-1.49)	0.527
	T/T	1	1	50.00	0.77 (0.05-12.5)	1.000

[a] PD, progressive disease; SD, stable disease; PR, partial response; CR, complete response.
[b] OR, odds ratio; CI, confidence interval.

Supplementary Table 4: The characteristics of the patients

Characters	Variable	n	Rate (%)
Gender	male	169	61.68
	female	105	38.32
Age (year)	≤60	128	46.72
	>60	146	53.28
Response	CR	15	5.47
	PR	107	39.05
	PD	63	22.99
	SD	89	32.48
Toxicity	Nausea and vomiting	82	29.93
	Myelosuppression	56	20.44
	Liver dysfunction	21	7.66
	Diarrhea	16	5.84
	Neurotoxicity	8	2.92
	Others	5	1.82
	No	86	31.39

Supplementary Table 5: The synthetic oligonucleotides used for construction of plasmids and qPCR

Gene	GenBank ID			Forward primer (5'→3')	Reverse primer (5'→3')
TYMS	NM_001071.2	CDS	qPCR	CTGCCAGCTGTACCAGAGAT	ATGTGCATCTCCCAAAGTGT
		Wild-type 3'-UTR	pGL3 construct	GACTAG<u>TCTAGA</u>GGTGCTTTCAAAGGAGCTCG	GGCTAG<u>GTTAAC</u>TCAAAGCCTTTTATTATGGA
		Mutant 3'-UTR	pGL3 construct	GGGGTTGGGCTGGATCGGCTCGTAAAAGTTCTTTTTGC	GCAAAAAGAACTTTTACGAGCCGATCCAGCCCAACCCC
ABCC3	NM_003786.3	3'-UTR	pGL3 construct	GGCTAG<u>TCTAGA</u>ATATTCCTGAGATTTCCTC	GGCTAG<u>GTTAAC</u>TTTACTGTTGACCATGTAG
UCK1	NM_031432.3	3'-UTR	pGL3 construct	GGCTAG<u>TCTAGA</u>TTTGGAGTCCAGCAGCAGAC	GGCTAG<u>GTTAAC</u>CTAGACAGACAGCACCTTGG
miR-1307-3p		mimics	Regulation	ACUCGGCGUGGCGUCGGUCGUG	CGACCGACGCCACGCCGAGUUU
		inhibitor	Regulation	CACGACCGACGCCACGCCGAGU	
		Stem-loop	qPCR	GTCGTATCCAGTGCAGGGTCCGAGGTATTCGCACTGGATACGACCACGAC	
			qPCR	CACTCGGCGTGGCGTCG	GTGCAGGGTCCGAGGT
GAPDH	NM_002046.5	mRNA	qPCR	TGCACCACCAACTGCTTAGC	GGCATGGACTGTGGTCATGAG
U6	NR_004394.1	ncRNA	qPCR	CTCGCTTCGGCAGCACA	AACGCTTCACGAATTTGCGT
