# Supplementary material for: Rs7911488 modified the efficacy of capecitabine-based therapy in colon cancer through altering miR-1307-3p and TYMS expression
Source: Oncotarget. 2017 Jul 28;8(43):74312–9. doi: 10.18632/oncotarget.19670 (PMC5650342; doi:10.18632/oncotarget.19670)
Supplement: Supplementary file 2 [file oncotarget-08-74312-s002.docx]

**Supplemental Materials**

**Table S1.** The polymorphisms in the pre-miRNAs and the predicted target genes

| SNP | Variation | MAF^a^ | miRNA | Predicted targets |
| --- | --- | --- | --- | --- |
| rs6505162 | C>A | A=0.4881 | miR-423-3p | ABCC5, CDA, UCK1 |
| rs2292832 | C>T | T=0.4351 | miR-149 | ABCC3, CDA, CES2, MTHFR, RRM2, UPB1, UMPS |
| rs2910164 | G>C | C=0.3812 | miR-146a | ABCC4, ABCC5, ABCG2, SLC22A7, SLC29A1, TK1, UPB1, UMPS, UPP2 |
| rs895819 | A>C | C=0.3569 | miR-27a | ABCC4, CES2, DPYD, DPYS, MTHFR, PPAT, RRM2, UCK2, UCK1, UMPS, UPB1, UPP1, UPP2 |
| rs7911488 | T>C | C=0.2674 | miR-1307-3p | TYMS, UCK1, ABCC3 |
| rs12402181 | G>A | A=0.2639 | miR-3117 | ABCG2 |
| rs28599926 | C>T | T=0.2002 | miR-1268 | ABCG2, MTHFR, RRM2, TK1, UCK1 |
| rs3746444 | T>C | C=0.1819 | miR-499-3p | ABCG2, TYMS, UCK2 |
| rs12416605 | C>T | T=0.1527 | miR-938 | DPYS, UPB1 |
| rs71428439 | A>G | G=0.1435 | miR-149 | ABCC3, CDA, CES2, MTHFR, RRM2, UPB1, UMPS |
| rs2620381 | U>G | C=0.0841 | miR-627 | MTHFR, CES2, ABCC4, PPAT, UCK1, DPYS |
| rs78825966 | C>T | T=0.0599 | miR-557 | MTHFR, UPP2 |
| rs76481776 | G>A | T=0.0484 | miR-182 | ABCC4, SLC22A7, DPYD, MTHFR, PPAT, RRM2, UCK2 |
| rs9745376 | G>A | A=0.0466 | miR-662 | UCK1, RRM2 |
| rs12220909 | C>G | C=0.0402 | miR-4293 | MTHFR |
| rs116838571 | A>U | A=0.0397 | miR-3689b | ABCG2 |
| rs35544770 | G>A | A=0.0297 | miR-941 | ABCC3, SLC29A1, UCK1 |
| rs11973069 | C>U | T=0.0283 | miR-4284 | ABCC4 |
| rs6971711 | C>T | T=0.0274 | miR-590-5p | ABCC5, PPAT, UMPS, UPP2 |
| rs74647838 | C>T | A=0.0251 | miR-1302 | MTHFR, UCK1, UMPS |
| rs113297757 | G>A | A=0.0224 | miR-3196 | CDA |
| rs72631825 | G>A | A=0.019 | miR-222 | ABCC5, ABCG2, SLC29A1, MTHFR, RRM2, TK1, UPP2 |
| rs114399468 | G>A | A=0.0155 | miR-4322 | CES2, TK1, PPAT, UMPS |
| rs11231898 | C>T | A=0.0137 | miR-194 | ABCG2, PPAT, UCK1, UMPS |
| rs74743733 | G>A | A=0.0133 | miR-4257 | ABCC3, SLC29A1, TK1, MTHFR, PPAT |
| rs73159662 | C>T | A=0.01 | miR-96 | ABCC4, DPYD, MTHFR, PPAT, RRM1, RRM2, UCK2 |
| rs12081872 | C>U | T=0.0091 | miR-3124 | ABCC5 |
| rs72631826 | T>C | G=0.0087 | miR-16 | ABCC3, ABCC5, SLC22A7, MTHFR, RRM2, TK1, TYMS, UPB1 |
| rs41280052 | G>T | T=0.0059 | miR-184 | ABCC4, CES2, MTHFR, RRM2, UCK1 |
| rs116476604 | U>C | G=0.0059 | miR-466 | ABCC4 |
| rs11671784 | C>T | A=0.0059 | miR-27a | ABCC4, CES2, DPYD, DPYS, MTHFR, PPAT, RRM2, UCK2, UCK1, UMPS, UPB1, UPP1, UPP2 |
| rs6122014 | G>A | A=0.0055 | miR-1 | ABCC4, ABCC5, DPYS, MTHFR, RRM2, UMPS, UPB1 |
| rs41274312 | C>T | A=0.0055 | miR-187 | ABCC3, ABCC4, PPAT, UMPS |
| rs72631828 | A>G | G=0.005 | miR-10a | ABCC3, ABCG2, MTHFR, RRM2, TK1, UCK1, UMPS |
| rs72563729 | G>A | A=0.005 | miR-200b | ABCG2, MTHFR, PPAT, RRM2 |
| rs72631824 | C>T | A=0.005 | miR-93 | ABCC5, ABCG2, CES2, DPYD, MTHFR, PPAT, RRM2, TK1, UPP1 |
| rs41292412 | C>T | T=0.0046 | miR-122 | ABCC5, ABCG2, SLC22A7, CDA, CES2, MTHFR, RRM2, TK1 |
| rs72631833 | G>T | A=0.0046 | miR-183 | ABCC4, ABCC5, ABCG2, SLC22A7, CES2, DPYS, MTHFR, PPAT, RRM2, UCK1, UCK2, UMPS, UPP1 |
| rs114964240 | G>A | T=0.0037 | miR-187 | ABCC3, ABCC4, PPAT, UMPS |
| rs113212828 | A>G | G=0.0037 | miR-605 | RRM2, ABCC4, PPAT |
| rs72631822 | G>A | A=0.0037 | miR-130b | ABCC3, ABCC5, DPYD, MTHFR, RRM2, UPP2 |
| rs41274239 | T>C | G=0.0032 | miR-96 | ABCC4, DPYD, MTHFR, PPAT, RRM1, RRM2, UCK2 |
| rs117258475 | C>T | A=0.0032 | miR-296-3p | ABCC4, SLC22A7, MTHFR, PPAT, RRM2, UPP2 |
| rs80041074 | C>T | T=0.0027 | miR-182 | ABCC4, SLC22A7, DPYD, MTHFR, PPAT, RRM2, UCK2 |
| rs73602910 | G>A | A=0.0027 | miR-518d-3p | ABCC4, TYMS |
| rs72631827 | G>T | A=0.0023 | miR-106b | ABCC5, ABCG2, CES2, DPYD, MTHFR, PPAT, RRM2, UPP1 |
| rs111424617 | C>T | T=0.002 | miR-30b | ABCC4, ABCC5, DPYD, DPYS, PPAT |
| rs77586312 | C>T | A=0.0014 | miR-182 | ABCC4, SLC22A7, DPYD, MTHFR, PPAT, RRM2, UCK2 |
| rs72631835 | C>T | A=0.0014 | miR-199b-5p | ABCC3, ABCC5, ABCG2, SLC22A7, MTHFR, RRM2, TK1, TYMS, UCK1, UMPS |
| rs75953509 | A>G | C=0.001 | miR-182 | ABCC4, SLC22A7, DPYD, MTHFR, PPAT, RRM2, UCK2 |
| rs72631834 | T>C | G=0.0009 | miR-215 | ABCC3, ABCG2, CES2, DPYD, TYMS, UCK1, UPP2 |
| rs72631823 | G>A | T=0.0005 | miR-34a | ABCC4, ABCC5, SLC22A7, SLC29A1, CDA, DPYD, MTHFR, RRM2, TK1, UMPS |
| rs78902025 | T>G | G=0.0005 | miR-642a | MTHFR, DPYS, RRM2 |
| rs35301225 | G>T | NA | miR-34a | ABCC4, ABCC5, SLC22A7, SLC29A1, CDA, DPYD, MTHFR, RRM2, TK1, UMPS |
| rs118009022 | T>G | NA | miR-195 | ABCC3, ABCC5, SLC22A7, MTHFR, RRM2, TK1, TYMS, UPB1 |
| rs60406007 | G>T | NA | miR-193a-5p | CES2, MTHFR, RRM2, UPB1 |
| rs113054794 | G>T | NA | miR-221 | ABCC5, ABCG2, SLC29A1, MTHFR, RRM2, TK1, UPP2 |
| rs112599381 | T>C | NA | miR-133b | ABCC5, SLC22A7 |
| rs112062096 | T>C | NA | miR-204 | SLC22A7, CES1, CES2 |
| rs34952329 | insC | NA | miR-223 | ABCC5, SLC29A1, MTHFR, PPAT, RRM2, UCK1, UMPS |
| rs35143473 | T>del | NA | miR-365 | ABCC4, ABCG2, SLC29A1, DPYD, MTHFR, PPAT, RRM2, UPP2 |
| rs41274221 | G>A | NA | miR-25 | ABCC5, DPYD, PPAT, UPB1 |
| rs41281222 | G>A | NA | miR-183 | ABCC4, ABCC5, ABCG2, SLC22A7, CES2, DPYS, MTHFR, PPAT, RRM2, UCK1, UCK2, UMPS, UPP1 |
| rs113859371 | C>T | NA | miR-205 | ABCC4, ABCC5, ABCG2, SLC22A7, CES1, DPYD, DPYS, MTHFR, PPAT, RRM2, UPB1 |
| rs7266947 | T>G | NA | miR-663 | MTHFR, RRM2, UCK1 |
| rs41265488 | T>A | NA | miR-9 | ABCC4, ABCC5, SLC22A7, MTHFR, PPAT, RRM2, UCK1, UMPS |
| rs112971695 | T>C | NA | miR-204 | SLC22A7, CES1, CES2 |
| rs113322127 | T>C | NA | miR-135a | ABCG2, CES2, MTHFR, PPAT, RRM2, UMPS, UPP2 |
| rs41275866 | C>G | NA | miR-18a | ABCC3, ABCC5, CDA, CES2, DPYD, MTHFR, RRM2, TK1, UPP1 |
| rs28670321 | A>G | NA | miR-663 | MTHFR, RRM2, UCK1 |
| rs113749278 | T>C | NA | miR-30c | ABCC4, ABCC5, DPYD, DPYS, PPAT |
| rs34059726 | G>T | NA | miR-124 | ABCC3, SLC29A1, UCK1, TYMS |
| rs113283070 | C>G | NA | miR-941 | MTHFR |
| rs13276615 | G>U | NA | miR-3622b-5p | ABCC5 |
| rs4636784 | G>C | NA | miR-4305 | ABCG2, UCK1, RRM2 |
| rs7167371 | C>G | NA | miR-3118 | UCK1, UPB1 |
| rs111664333 | C>T | NA | miR-642b | DPYD, ABCG2, UCK1 |
| rs76084273 | C>A | NA | miR-3910 | ABCC5 |
| rs12975333 | G>T | NA | miR-125a-5p | RRM2 |
| rs11382316 | insA | NA | miR-3161 | MTHFR, ABCC4, DPYD |
| rs113098367 | insA | NA | miR-3161 | MTHFR, ABCC4, DPYD |
| rs61786895 | G>A | NA | miR-3118 | ABCG2, UPB1, UPP2, DPYD |
| rs11488501 | G>A | NA | miR-3118 | ABCG2, UPB1, UPP2, DPYD |
| rs34381260 | insA | NA | miR-1276 | ABCG2, ABCC5 |
| rs76857625 | A>G | NA | miR-1304 | UCK1, PPAT |
| rs113672516 | G>A | NA | miR-941 | ABCC3, SLC29A1, UCK1 |
| rs75823810 | G>A | NA | miR-3939 | ABCG2 |
| rs118080115 | A>G | NA | miR-3614-5p | ABCC3, ABCG2 |
| rs13276615 | A>C | NA | miR-3622b-5p | MTHFR |
| rs13276615 | A>C | NA | miR-3622b-5p | UCK1, MTHFR |
| rs12159555 | C>G | NA | miR-3618 | RRM2 |

^a^MAF, minor allele frequency in the 1000 Genomes project (http://www.internationalgenome.org/).
